# Supplementary material for: Measurement of Intermediate Frequency Magnetic Fields Generated by Household Induction Cookers for Epidemiological Studies and Development of an Exposure Estimation Model
Source: Int J Environ Res Public Health. 2022 Sep 21;19(19):11912. doi: 10.3390/ijerph191911912 (PMC9565691; doi:10.3390/ijerph191911912)

Supplementary Material Appendix S1

Used Questionnaire at Phase 2 survey

Translated from Japanese to English

Q.1

Referring to the diagram below, measure the actual distance between (1) and (2) for each of your cookers.

Please use the enclosed measuring tape for measurement.
Please be sure to check that the fire is completely extinguished and that it is safe enough before taking measurements.

（1） Most frequently used
Distance from the center of the stove/heater circle to the front of the cooktop: Distance a cm

（2） **Distance from the front of the cooktop to the front of your abdomen： Distance** b cm

Reference chart

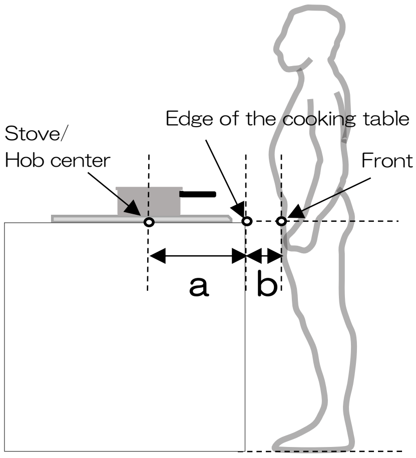

Side View

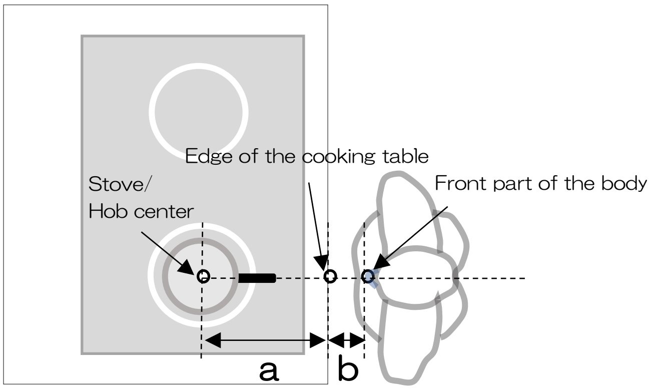

Top View

Q.2

We would like to ask you about the **coocware** you use in your cooker.
Please answer each of the following questions (1) through (3).

(1) Please circle one number that applies to the type of **cookware, you** use most often.

1 Frying pan	2 Kettles	3 Two Handed Pots
4 One-Handed Pots	5 Other ( )	

(2) Please circle one number that applies to the material of the pots and pans you use most frequently in (1).

1 iron	2 Enamel	3 Stainless steel	4 Aluminum
5 Tiphar	6 Other)		
7 Mixed material ( )	8 Unknown		

(3) Please indicate the diameter of the bottom of the most commonly used pots and pans that you answered in (1).
Please use the enclosed measuring tape to measure.

________cm

Reference chart

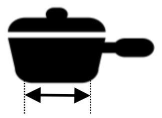

Diameter of cookware bottom

Q.3

Asking this question to those of you who use induction cooktops.

Please fill in the product information of your induction cooker.
Please check the front of housing or the user's manual before you answer.
↳ The appliance number and model number are located

Please check the label on the unit or the instruction manual before answering the question.

Manufacturer	1 Panasonic 2 Hitachi 3 Mitsubishi<br>4 Other ( )
Appliance number / Model number	( )
Year of manufacture	Year:

Reference chart

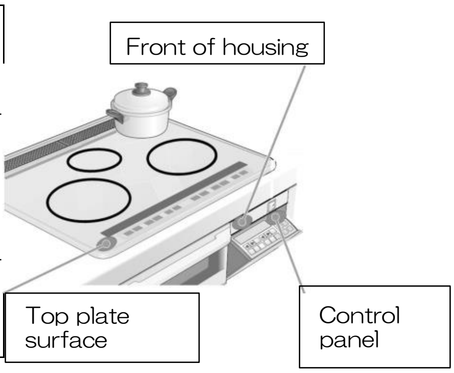

Supplement: Supplementary file 1 [file ijerph-19-11912-s001.zip › ijerph-1900138-supplementary.pdf]
